# Transcriptome Analysis of Chemically-Induced Sensory Neuron Ablation in Zebrafish

**DOI:** 10.1371/journal.pone.0148726

**Published:** 2016-02-10

**Authors:** Jane A. Cox, Bo Zhang, Holly M. Pope, Mark M. Voigt

**Affiliations:** 1 Department of Pharmacology and Physiology, Saint Louis University School of Medicine, St. Louis, MO, United States of America; 2 Center for Neuroscience, Saint Louis University School of Medicine, St. Louis, MO, United States of America; 3 Center for Regenerative Medicine, Department of Developmental Biology, Washington University School of Medicine, St. Louis, MO, United States of America; Federal University of Rio de Janeiro, BRAZIL

## Abstract

Peripheral glia are known to have a critical role in the initial response to axon damage and degeneration. However, little is known about the cellular responses of non-myelinating glia to nerve injury. In this study, we analyzed the transcriptomes of wild-type and mutant (lacking peripheral glia) zebrafish larvae that were treated with metronidazole. This treatment allowed us to conditionally and selectively ablate cranial sensory neurons whose axons are ensheathed only by non-myelinating glia. While transcripts representing over 27,000 genes were detected by RNAseq, only a small fraction (~1% of genes) were found to be differentially expressed in response to neuronal degeneration in either line at either 2 hrs or 5 hrs of metronidazole treatment. Analysis revealed that most expression changes (332 out of the total of 458 differentially expressed genes) occurred over a continuous period (from 2 to 5 hrs of metronidazole exposure), with a small number of genes showing changes limited to only the 2 hr (55 genes) or 5 hr (71 genes) time points. For genes with continuous alterations in expression, some of the most meaningful sets of enriched categories in the wild-type line were those involving the inflammatory TNF-alpha and IL6 signaling pathways, oxidoreductase activities and response to stress. Intriguingly, these changes were not observed in the mutant line. Indeed, cluster analysis indicated that the effects of metronidazole treatment on gene expression was heavily influenced by the presence or absence of glia, indicating that the peripheral non-myelinating glia play a significant role in the transcriptional response to sensory neuron degeneration. This is the first transcriptome study of metronidazole-induced neuronal death in zebrafish and the response of non-myelinating glia to sensory neuron degeneration. We believe this study provides important insight into the mechanisms by which non-myelinating glia react to neuronal death and degeneration in sensory circuits.

## Introduction

In the peripheral nervous system, damage to and/or loss of sensory neurons can result in debilitating neuropathies [1–7] that often have a dramatic impact on quality of life. The cellular mechanisms involved in the response of neurons and glia to such pathological insults are poorly understood. Identification of the genes involved in the regulation and dysregulation of these pathways could offer the promise of new therapeutic approaches to treating these disorders.

Investigations into the pathophysiology of neuronal damage/death typically have utilized experimental paradigms involving non-selective damage to peripheral nerves; e.g., constricting, crushing or cutting nerves [8–10] or using non-targeted pharmacological agents [11, 12]. Such studies have shown that peripheral glia play critical roles in both the degenerative and regenerative processes that are involved in the responses to peripheral nerve damage [13–15]. Most efforts have focused primarily on myelinating Schwann cells [15–18], with the result that very little is known regarding how the non-myelinating glia that ensheath axons and neuronal somas respond to nerve damage. This is a significant knowledge gap given that approximately 80% of cutaneous nerve fibers are unmyelinated [19, 20], that they transduce such important modalities as itch, pain, temperature and mechanosensation [21–25], and that they are affected in many prevalent peripheral neuropathies [26].

Previous work from this lab has shown that the peripheral sensory circuits in the head are formed in zebrafish by 4 dpf [27]. At this stage, the neurons in the cranial ganglia are encapsulated by satellite glia and their axons are ensheathed by peripheral glia. Unlike the lateral line nerves of the head and trunk, the trigeminal and epibranchial circuits are unmyelinated at 4 dpf and remain so until at least 10 dpf [28]. Thus the ensheathing glia of these circuits are non-myelinating Schwann cells. In a recent report, we described a zebrafish line that allows for the selective ablation of these unmyelinated cranial nerves using the metronidazole (MET)-*nsfB* cell-death system [29]. Upon exposure to MET, the cranial neurons selectively expressing the *nsfB* transgene quickly died, with the affected cell bodies and axons degenerating over the course of 18 hours of treatment. This model therefore offers an opportunity to investigate the response of non-myelinating glial to nerve degeneration without the confounding contributions of myelinating Schwann cells. Of particular relevance to the current study is the fact that these neurons, and their unmyelinated axons, are restricted to the head.

The organismal response to loss of the affected sensory neurons and their axons was composed of several phases. The first phase, occurring in the first 5 hours of treatment, consisted of increased cell death followed closely by the beginnings of axonal degeneration. The second phase, occurring between 5–18 hours of treatment, comprised phagocytosis of the cell body/axonal debris and recruitment of macrophages to the areas of degeneration. Further analysis using *foxd3*^*zd10*^ mutants, which lack peripheral glia, demonstrated that both phagocytosis of axonal/neuronal debris and recruitment of immune cells were dependent on the presence of the non-myelinating glia ensheathing the neuronal somas and axons [29].

In this report, we utilized RNA-seq to identify genes that were differentially expressed in the larval head during the process of sensory neuron ablation and axon degeneration. Transcriptome analysis revealed that MET-induced toxicity affected only a small subset of genes (a total of 458, which is <1% of the total number found to be expressed in the head at this stage). Significant changes in gene expression reflected alterations in a select number of cellular pathways: e.g., membrane transport, anti-inflammatory pathways, oxidative stress, metabolism and cytokine signaling. Analyses were also carried out using RNA isolated from mutant larvae (*foxd3*^*zd10*^ nulls) that lack all peripheral glia [29–31]. In those experiments, no significant effect was seen on pathways involved in cytokine signaling, response to stress and oxidoreductase activities. Overall, the information regarding differential gene expression in these conditions provide a basis for further investigation into the cellular processes that underlie pathophysiological responses of neurons and glia to sensory nerve damage.

## Materials and Methods

### Ethics Statement

All experiments were carried out in accordance with the National Institutes of Health Guide for the Care and Use of Laboratory Animals. The Saint Louis University Animal Care Committee approved the procedures used (protocol # 1619) and all efforts were made to minimize the number of animals used and their suffering.

### Maintenance of fish and fish strains

All animal husbandry was carried out as described by Westerfield [32], and staging was carried out using the criteria of Kimmel et al. [33]. For some experiments, reduction of pigmentation of embryos/larvae was achieved by growing embryos in fish water containing phenylthiourea (PTU) at a concentration of 0.0015%. The following strains were used: *Tg(p2rx3*.*2(-4*.*0)*:*gal4vp16)*;*Tg(UAS*:*nsfB-mcherry)*, *Tg(p2rx3*.*2(-4*.*0)*:*gal4vp16)*;*Tg(UAS*:*nsfB-mcherry)*:*csfr1a*^*j4e1*^ and *Tg(p2rx3*.*2(-4*.*0)*:*gal4vp16)*;*Tg(UAS*:*nsfB-mcherry)*:*foxd3*^*zd10*^ [29], and *Tg(p2rx3*.*2(-4*.*0)*:*gal4vp16)*;*Tg(kaede)* [34].

### Metronidazole treatment

Larvae at 4dpf were treated with metronidazole (Sigma, St. Louis, MO) at a final concentration of 10 mM in fish water for either 2 or 5 hours. Control fish were treated with fish water alone. Verification of neuron ablation was carried out using epifluorescent imaging as described in [29].

### RNA sequencing and bioinformatic analyses

Heads of 4 dpf larvae exposed to 10 mM MET for either 0, 2 or 5 hours were dissected using a small scalpel (Katena Products Inc.), with 8 heads pooled per sample. Each sample was then homogenized in 250 μL of TRIzol (Life Technologies), and total RNA extracted. The RNA samples were then treated with DNase and column purified using a Qiagen RNEasy mini kit (Qiagen, Inc.). Biological triplicates were prepared for each condition. At this point, the total RNA samples were supplied to the Genome Technology Access Center at Washington University in St. Louis for library construction and high-throughput sequencing. RNA purity and integrity was determined using an Agilent Bioanalyzer 2100, and only those samples having a RIN > 8.7 were used. For library construction, 1 μg total RNA was used for each wild-type replicate and 0.35 μg for the mutant replicates. The total RNA samples were depleted of rRNA using the Ribo-Zero protocol (Epicentre). cDNA was synthesized, blunt ended, an A base added to the 3’ ends and Illumina sequencing adapters ligated using a proprietary protocol with enzymes from Enzymatics (Qiagen). cDNA fragments were then amplified for 12 cycles using primers incorporating unique index tags and multiplexed sequencing was performed using an Illumina HiSeq2500 platform (single end, 50 bp reads).

RNA-seq reads were aligned to the Danio rerio Zv9 assembly from Ensembl with STAR [35] version 2.0.4b. Gene counts were derived from the number of uniquely aligned unambiguous reads by Subread:featureCount [36], version 1.4.5. Transcript counts were produced by Sailfish [37], version 0.6.3. Sequencing performance was assessed for total number of aligned reads, total number of uniquely aligned reads, genes and transcripts detected, ribosomal fraction, known junction saturation and read distribution over known gene models with RSeQC [38] version 2.3. All gene-level and transcript counts were then imported into the R/Bioconductor package EdgeR [39] and TMM normalized to adjust for differences in library size. Genes or transcripts not expressed in any sample were excluded from further analysis. Performance of the samples was assessed with a Spearman Correlation Matrix (S1 Fig). The data generated in this study has been deposited in the National Center for Biotechnology Information Gene Expression Omnibus (GEO) database (accession number GSE 72682). Generalized linear models with robust dispersion estimates were created to test for gene/transcript level differential expression. The fit of the trended and tagwise dispersion estimates were then plotted to confirm proper fit of the observed mean to variance relationship where the tagwise dispersions are equivalent to the biological coefficients of variation of each gene. Differentially expressed genes and transcripts were then filtered for those having fold-changes (FC) > 1.2 together with false-discovery rate (FDR) adjusted p-values less than or equal to 0.05. R/Bioconductor package “gplots” was used to generate the heatmaps. The differentially expressed zebrafish genes in each condition were transferred to their human orthologs by using the PANTHER classification system (http://pantherdb.org/genes/index.jsp), then GO enrichment analysis was performed in ToppGene suite [40], with the p-value cut-off < 0.05 (corrected using the Benjamini and Hochberg (B&H) procedure).

A primary consideration in performing GO analysis was how to organize the identified DEGs into the requisite sets. Given that a relatively small population of cells in the head would be expected to be directly affected by MET treatment (peripheral sensory neurons, the peripheral glia that ensheath them, and a small number of *3*.*2*:*NTR*^*+*^ retinal cells), there was some concern that information might be lost if the pool of genes analyzed were chosen using too stringent of cutoffs. Also, since the exposure to MET was continuous over the 5 hrs, we also wanted an approach that would treat the data as dynamic rather than static. And most importantly, it was critical to have confidence in the analyses. Therefore, the following analytic strategy was used. First, a master pool which contained only the genes that met stringent statistical cutoffs (in our case, B&H derived FDR<0.05 and FC >1.2 or <-1.2) at either 2 hrs, 5 hrs, or at both time points was formed. Next, the FC values for each of the genes in the pool which met both cutoff values at one time point only were evaluated as a means to identify DEGs that were significant at one time point but whose expression was trending either up (FC > 1.2) or down (FC<1/1.2) at the time at which their FDR >0.05 (non-significant). Based on those two sets of criteria, it was possible to segregate the DEGs into one of three categories- 1) early response: contains DEGs which met the FDR and FC cutoffs at 2 hrs but not at 5 hrs, 2) continuous response: contains the sets of DEGs that met the FDR and FC criteria at both time points *plus* those that met the FDR and FC cutoffs at one time point but only the FC at the second time point, and 3) late response: those DEGs which met the FC and FDR criteria at 5 hrs but not at 2 hrs.

### Quantitative PCR

Total RNA was extracted from different experimental animals than those used for the transcriptome analysis using the protocol detailed above. Oligo-dT primed cDNA was then synthesized using a Superscript III first-strand synthesis kit (Life Technologies, INC), and qPCR carried out using validated prime sets obtained from SA Bioscience (Qiagen, Inc). For the qPCR, 1 μL of cDNA was used in a 10 μL SYBR green reaction mix. PCR and post-reaction analysis was carried out using a StudioQuant 6 PCR machine (Life Technologies, Inc).

### Analysis of Neurogenesis

Previous work by others have shown that the photoconvertible protein kaede can be used as a tool in neuronal birthdating [41]. Kaede is a green fluorescent protein that is converted into a stable red-fluorescent fragment after exposure to UV light. Given that the cranial sensory ganglia consist of tightly-packed clusters of neurons, we chose an approach that would allow us to obtain labeling of only a small number of neurons in each ganglion, so that we could reliably quantitate labeled cell bodies. To accomplish this, we obtained variegated expression of kaede in the epibranchial neurons using a binary expression system (*p2rx3*.*2*^*−4*.*0*^:*gal4VP16;UAS*:*kaede*) [34]. On the first day (3, 4, 5 or 7 dpf), photoconversion was carried out on live embryos/larvae that had been anesthetized with tricaine (0.01%). Conversion was performed using a 20x water immersion objective mounted on an Olympus FV-1000 MPE confocal microscope, with the region of interest (ROI) being the area containing the epibranchial ganglia. Before photoconversion was performed, a scan was done to verify that there was no pre-existing red-fluorescence within the ROI. Next, while imaging the target area in both the red and green channels, the 405nm laser was applied at a power of 0.5% or 1% for up to 1 minute, resulting in nearly all green fluorescence disappearing while robust red fluorescence appeared in every kaede^+^ neuron. The embryos/larvae were then washed 3x with fish water and returned to the fish facility. After 24 hours, the photoconverted embryos/larvae were then imaged again in both the green and red channels, with z-stacks being obtained. All z-planes were collapsed into maximal intensity projections using either Olympus Fluoview or NIH ImageJ software. Numbers of green^+^/red^-^ and green^+^/red^+^ neurons were counted in each z-stack, with 4–11 embryos/larvae examined per time point studied. Final brightness and/or contrast values of images were adjusted in Adobe Photoshop CS4.

## Results and Discussion

Our working hypothesis is that alterations in gene expression underlie the morphological responses that are seen with metronidazole (MET)-induced cranial neuron ablation and axon degeneration in this transgenic line. To test this, we analyzed the transcriptome of 4 dpf zebrafish larval heads after two and five hours of MET treatment. Two hours was chosen as our previous work demonstrated that at this point sensory neurons had begun to die and the most distal aspect of nsfB-mCherry^+^ axons were beginning to show structural alterations at both the light and electron microscopic levels. Five hours of MET exposure was chosen as by this time additional neurons were dying, phagocytosis of the sensory axons along their entire lengths had begun and recruitment of immune cells was initiated.

We chose to use the larval head as our RNA source for several reasons: first, there are no currently available zebrafish lines that allow for neuron or peripheral glia-specific transcriptome analysis, and second, at this stage the projections of the affected cranial sensory neurons are almost entirely restricted to the head. Therefore, using larval heads would ensure that we would capture any gene expression changes occurring in the targeted neurons, their ensheathing glia and, potentially, any cells undergoing sensory denervation as well. Sequencing to a depth greater than 30 million reads was carried out to ensure good representation of transcriptional changes that could occur in such a complex tissue (containing cells from dermis, muscle, nerves, ganglia, cartilage, mesenchyme, etc).

RNA was isolated from triplicate biological samples from 4 dpf *3*.*2*:*NTR* larvae treated with MET for either 0, 2 or 5 hours and subjected to RNA-seq. The transcriptome at two hours was sequenced to a depth of ~ 35 million reads/replicate, with ~94% of reads being uniquely mapped to the genome. This resulted in ~21 million gene counts per sample, which translates to an average of 27,098 genes detected per biological replicate. As the Zv9 genome assembly at Ensembl [42] predicts a total of 33,737 genes, our sequencing revealed that at this age the tissues in the head of the larvae expressed at least 81% of all predicted genes found in the zebrafish genome. Samples for the 5 hour time point were also sequenced to a depth of ~ 35 million reads. 95% of those mapped uniquely to the genome and gene counts revealed a total of 27,169 genes were detected. Since both sets of sequences yield equivalent depths of reads and coverage, analyses of the data was expected to provide reliable results. A detailed summary of the sequencing results is given in S1 Table.

### Differential Gene Expression

When the 2 hour transcriptome was compared to that of untreated controls, a total of 273 unique differentially expressed genes (DEGs) were detected (S2 Table). Of these, 179 were up-regulated and 94 down-regulated. The 10 transcripts in each group showing the greatest fold-changes are shown in Table 1. When RNA biotype was considered, there were 261 protein-coding RNAs, four long non-coding RNAs and eight processed transcripts (originating from coding genes, they are spliced but contain no open reading frame).

**Table 1:** Transcripts showing greatest fold change at 2 hr met treatment.

GENE NAME	DESCRIPTION	Log FC
**UP-REGULATED**		
CR788316.1	Uncharacterized protein, similar to verrucotoxin beta subunit	8.32766
CR788316.2	Uncharacterized protein, similar to neoverrucotoxin beta subunit	7.7638
si:ch73-313h21.1	processed transcript	4.99491
novel gene 100034660	Uncharacterized protein, endopeptidase S1 family	4.79187
asb11	ankyrin repeat and SOCS box-containing 11	3.31716
slc2a9l1	facilitated glucose transporter	2.26845
trim35-27	tripartite motif containing 35–27	1.86052
cx44.2	connexin 44.2	1.8152
tat	tyrosine aminotransferase	1.80159
cyp24a1	cytochrome P450, family 24, subfamily A, polypeptide 1	1.77717
**DOWN-REGULATED**		
si:ch1073-133p4.2	lincRNA	-4.87545
BX470200.1	Uncharacterized protein	-4.74224
ENSDARG00000074250	homeobox-containing unknown protein	-4.55278
gck	glucokinase (hexokinase 4, maturity onset diabetes of the young 2)	-3.63392
CR846087.1	Uncharacterized protein	-2.89839
nr0b2b	nuclear receptor subfamily 0, group B, member 2b	-2.85148
si:dkey-220o5.5	actin filament associated protein 1-like 2	-2.82413
zgc:172053	zgc:172053	-2.76296
si:dkey-25e11.13	processed transcript	-2.6941
si:dkey-31b16.11	processed transcript	-2.504

Analysis of the transcriptome at 5 hours of MET treatment, compared to that of controls, revealed a total of 299 unique DEGs (S3 Table). Of these, 236 were up-regulated and 63 down-regulated. The 10 transcripts in each group showing the greatest fold-change are also shown in Table 2. When RNA biotype was considered, there were 284 protein coding RNAs, four long non-coding RNAs, nine processed transcripts (spliced but containing no open reading frame), one miRNA and one snoRNA.

**Table 2:** Transcripts showing greatest fold change at 5 hr MET treatment.

GENE NAME	DESCRIPTION	Log FC
**UP-REGULATED**		
si:ch211-277g23.4	processed transcript	3.65999
si:dkey-29l4.4	lincRNA	3.25823
U3	Small nucleolar RNA U3	2.82427
slc2a9l1	facilitated glucose transporter	2.77719
CABZ01055869.1	uncharacterized protein, desmoglein 2-like	2.46025
cx44.2	connexin 44.2	2.36785
fosl1a	FOS-like antigen 1a	1.99903
si:ch211-120c15.3	uncharacterized protein, zinc-finger-like	1.98911
trim35-27	tripartite motif containing 35–27	1.91738
capn1	calpain 1	1.87402
**DOWN-REGULATED**		
ENSDARG00000074250	uncharacterized homeobox-containing protein	-4.51379
CABZ01034082.1	uncharacterized protein, zinc-finger	-3.72908
gck	glucokinase (hexokinase 4, maturity onset diabetes of the young 2)	-3.70692
si:dkey-228g21.5	processed transcript	-3.26187
CU207281.2	5-hydroxytryptamine 3A-like receptor	-2.63325
si:ch211-135n15.2	uncharacterized protein	-2.00013
zgc:174917	phytanoyl-coA dioxygenase domain-containing protein 1-like	-1.83274
zgc:111976	RNA polymerase II subunit 1-like	-1.79444
cyp2aa9	cytochrome P450, family 2, subfamily AA, polypeptide 9	-1.71989
si:ch73-334d15.4	potassium voltage-gated channel, Shaw-related subfamily, member 4-like (kcnc4-like)	-1.68789

For both time points, approximately 25–30% of affected genes could not be unambiguously identified. This was most probably due to either low sequence homologies with orthologous genes in other species, or that they represent unidentified teleost-specific genes. Those genes were not included in our analyses, as future studies will be needed in order to determine their identity and function.

Given that different processes were detectable by imaging at the two time periods examined, we expected the two DEGs panels would not be identical. Indeed, only 114 transcripts were shared between them, indicating that 159 genes present in the 2 hr DEGs pool were no longer different from control by 5 hours of treatment, and conversely, that 185 genes in the 5 hour pool had only become significantly altered after 2 hours of treatment. Of the 114 DEGs genes that were shared at 2 and 5 hours, 100 were up-regulated (vs. control) at both time points, with the other 14 DEGs down-regulated at both times.

The finding that only 1% of all genes detected in the samples showed differential expression at either time point revealed how discrete the effects of MET exposure were in the larval head. Also, the substantial differences in the DEG detected at either 2 or 5 hrs MET treatment indicate a dynamic change in gene expression that parallels the progressive organismal responses to MET-induced neuronal degeneration/death.

### Validation of results by quantitative PCR

**Fig 1:**
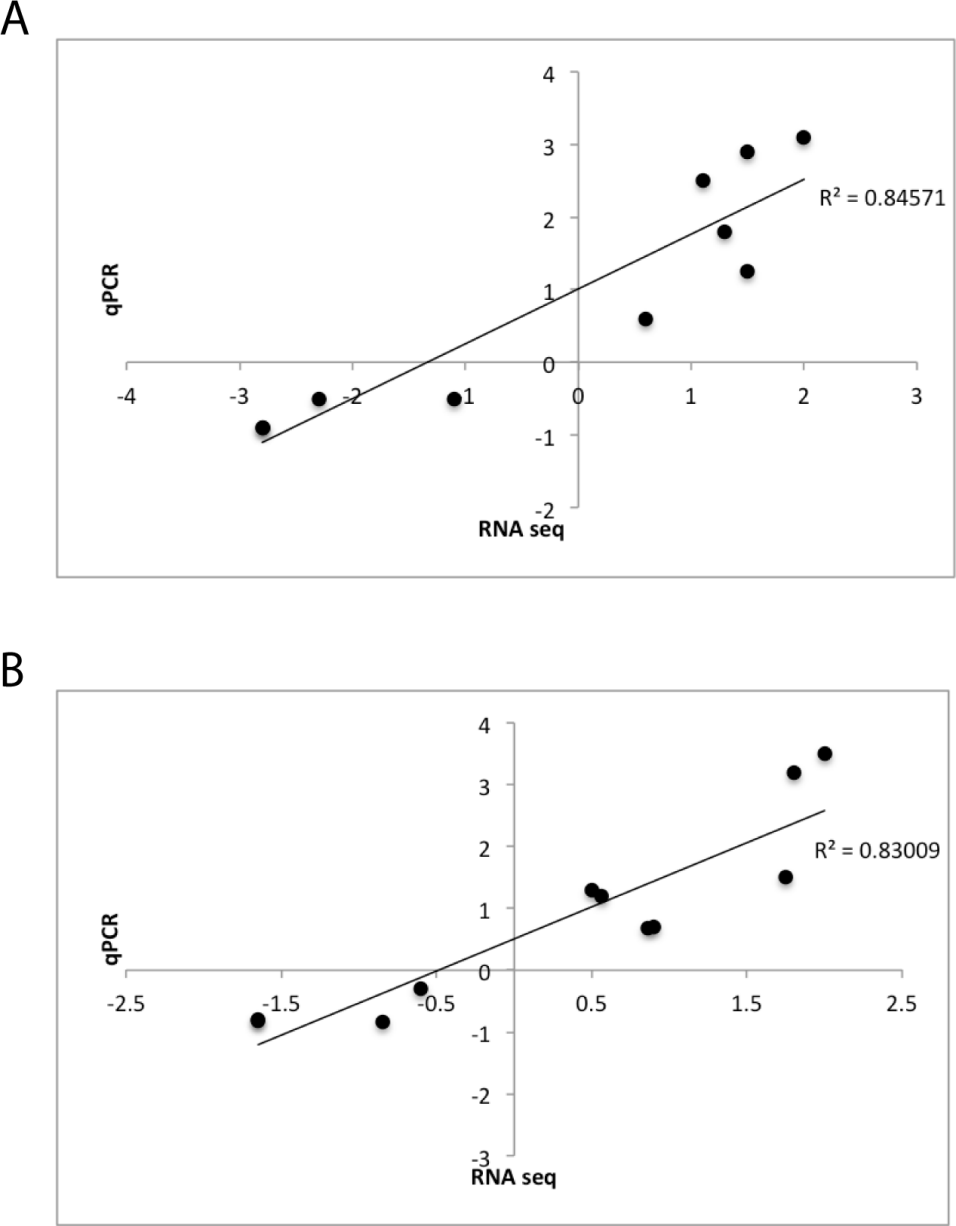
qPCR validation of representative DEGs at 2 and 5 hrs MET-treatment. Panel A: Plot of nine genes, found to be significantly affected after 2 hr of MET, showing RNAseq derived fold change (FC) (x-axis) vs. qPCR derived FC (ΔΔCt) (y-axis). Panel B: Plot of ten genes, found to be significantly affected after 5 hr of MET, showing RNAseq derived FC (x-axis) vs. qPCR derived FC (y-axis). Each point is the mean derived from three unique replicate biological samples. Linear regression (R^2^) values are shown inside each plot (p<0.001 for each panel).

To validate the expression profiles from the transcriptome analysis, we used qPCR to test the expression levels of targets that are differentially expressed at 2 hours only (*ifit2*, *cyp51*, *lpin1* and *trim35-30*), at 5 hours only (*grk5*, *gsr*, *il6st*, *junbb* and *tnfrsf1a*) and at both 2 and 5 hours (*cdca7a*, *nr02b2*, *cyp24a1*, *fosl1a*, and *tp53inp1*). Both up- and down-regulated genes are represented in these lists. As illustrated in Fig 1, the fold-change determined by qPCR for these genes showed strong and significant correlations to the values ascertained using RNAseq (for 2 hrs, r^2^ = 0.85, p<0.001; for 5 hrs, r^2^ = 0.83, p<0.001), findings which confirm the RNAseq results.

### Gene enrichment analysis of differentially expressed genes at 2 and 5 hours of MET-treatment

**Fig 2:**
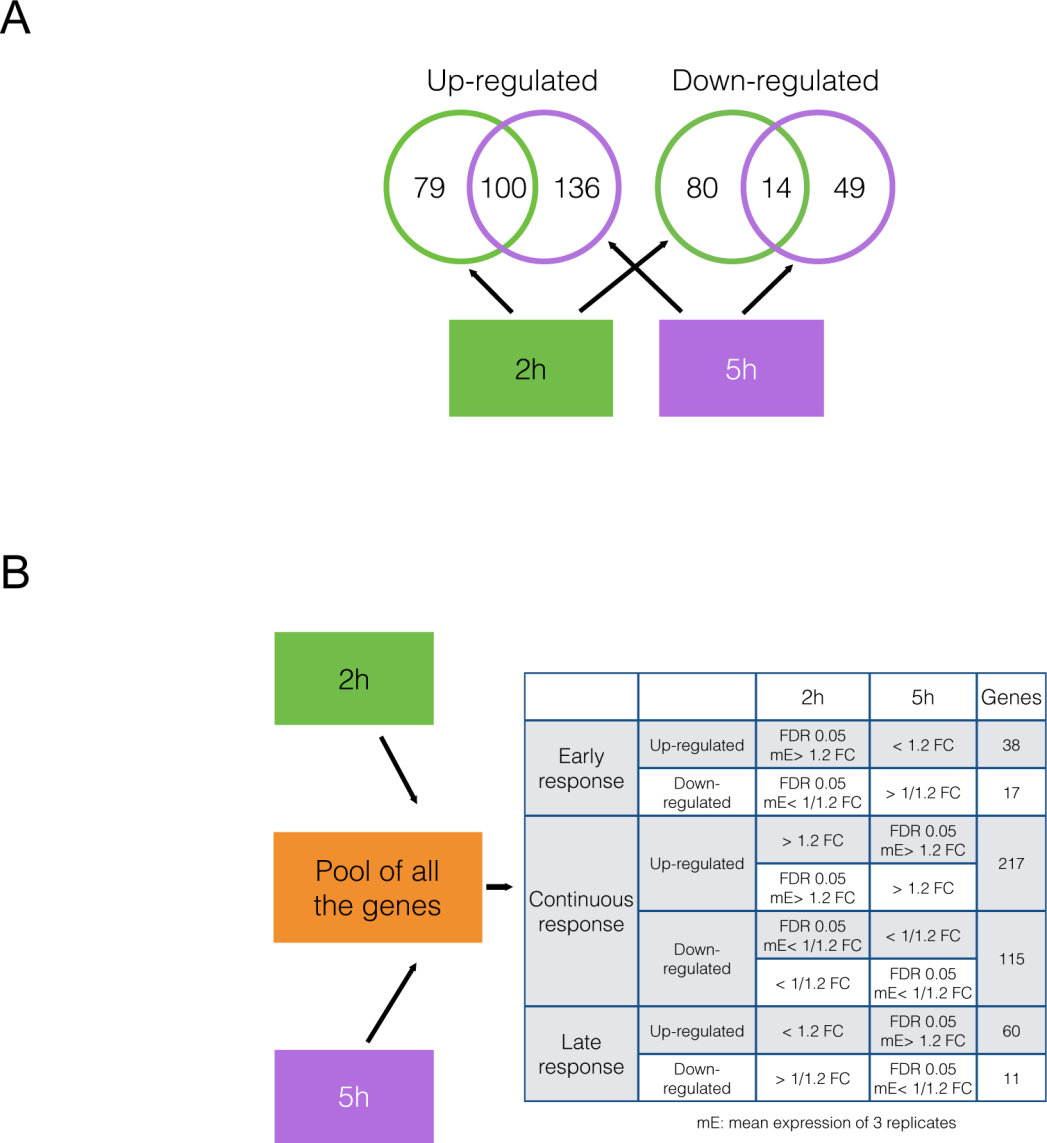
Methodological approach to analysis of DEGs identified during MET-induced sensory neuron ablation. Panel A: Venn diagram of up- and down-regulated DEGs present at 2 hrs (green) and 5 hrs (magenta) of MET exposure. The numbers of unique DEGs present either at one time point or found at both times are shown inside the circles. Panel B: Table listing the criteria for inclusion of genes shown in Panel A into one of three categories: early response, continuous response or late response. FDR = false-discovery rate (determined using Benjamini-Hochberg procedure); FC = fold-change.

Having identified genes showing differential expression at the two time points, our next step was to use gene enrichment analysis to gain insight into the biological, cellular and molecular processes potentially involved in the observed morphological responses. This was performed using GO (Gene Ontology) databases using the paradigm described in the Methods section. We were able to assign the 458 DEG (Fig 2A) into either the early response, continuous response or late response groups (Fig 2B).

**Fig 3:**
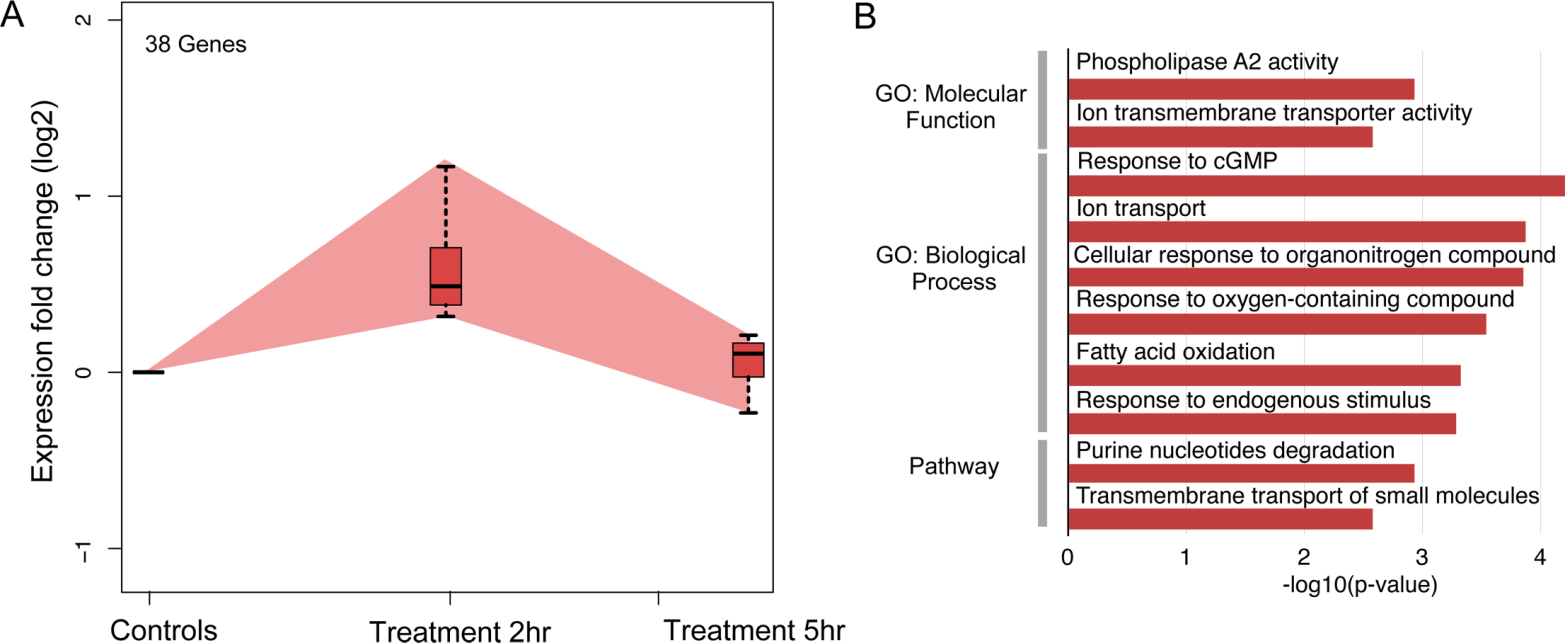
Analysis of genes found up-regulated only at 2 hrs of MET-treatment. Panel A: Plot of gene expression averaged from 3 biological replicates for those DEG found to only be up-regulated at 2 hrs of treatment. Panel B: Representative GO categories enriched for the up-regulated genes are shown with length of bar corresponding to degree of significance (longer bars = greater significance), *p*-values determined by Benjamini-Hochberg procedure.

The 55 DEG that were binned into the early response category could be further divided into 38 genes that showed up-regulation and 17 that were down-regulated. Due to this small number of DEGs, GO analysis resulted in only a few categories showing significant enrichment, and then only for the set of up-regulated DEGs; *e*.*g*. those involved in inflammation (*pnpla3* and *pl2g15*), membrane transport (*abcc5*, *slc7a2*, *slc12a7* and *slc38a4*) and responses to cyclic nucleotides (*pde3a* and *hcn4*) (Fig 3 and S4 Table).

**Fig 4:**
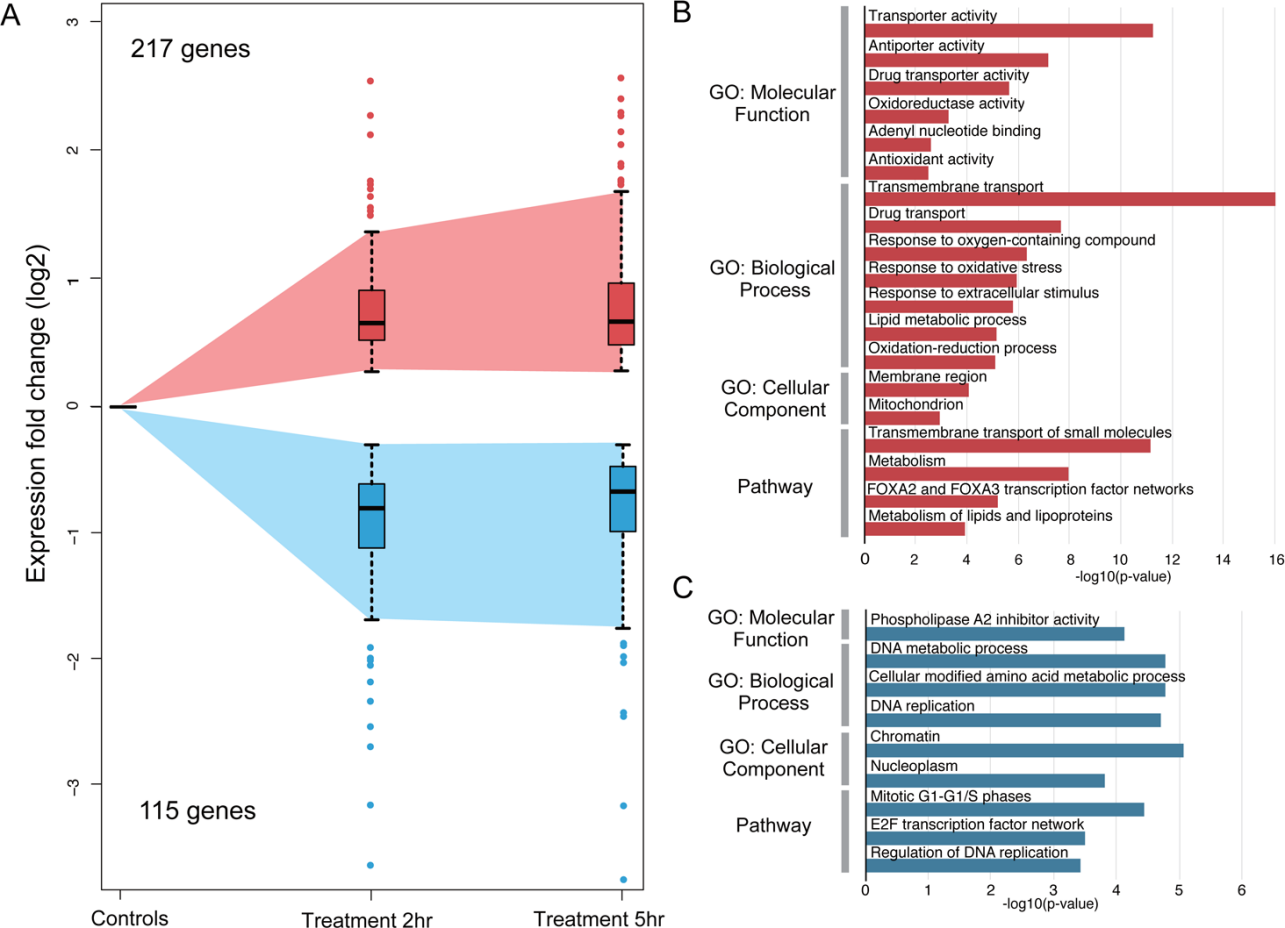
Analysis of DEGs present in the continuous expression group. Panel A: Plot of gene expression averaged from 3 biological replicates for those DEGs found to be up-regulated (red) or down-regulated (blue) between 2 and 5 hrs of MET treatment. Expression values beyond the means for each group at each time point are shown as single points either above or below the error bars. Panel B: Representative GO categories enriched for the up-regulated genes are shown. Panel C: Representative GO categories showing enrichment for the down-regulated genes are shown. For both Panels B and C, length of bar corresponds to degree of significance (longer bars = greater significance), *p*-values determined by Benjamini-Hochberg procedure.

With respect to DEGs that fell into the continuous category, 217 genes were found to show increased transcript numbers. The GO categories showing enrichment for these up-regulated genes (Fig 4 and S5 Table) included many for membrane transporter (37 DEGs) and metabolic activities (41 DEGs), processes that would be expected to be altered in the sensory neurons undergoing cell degeneration, as well as in supportive responses by the ensheathing glial cells. Additional categories with significant enrichment included oxidoreductase and oxidation-reduction activities, and the mitochondrion was scored as an affected cellular component. Given that metabolism of MET results in highly reactive cytotoxins [43], it is not unexpected that genes relevant to oxidative stress and mitochondrial function might be represented in the panel of DEGs. Indeed, 16 DEGs were found in the GO category for oxidoreductase activity (*abcc4*, *cyp24a1*, *cyp51a1*, *me1*, *pgd*, *dio3*, *hsd11b2*, *bbox1*, *hsdl2*, *tp53i3*, *tdo2*, *pir*, *gsr*, *loxl2*, *prdx1 and hsd17b12*) and 26 DEG mapped to the mitochondrion (*pdk2*, *pdk4*, *cyp24a1*, *slc25a25*, *me1*, *lpin1*, *slc25a43*, *ucp2*, *ucp3*, *agxt*, *slc25a38*, *bbox1*, *slc25a23*, *tat*, *hsdl2*, *sgk1*, *slc25a33*, *slmo2*, *abcb6*, *abcf2*, *cpt1b*, *gsr*, *arg2*, *gstp1*, *prdx1 and abcc12*). Notably, and consistent with the up-regulation of genes associated with mitochondrial function, the gene with one of the largest reductions in expression at both 2hr (FC = -13.2, FDR = 7.18e-5) and 5hr (FC = -13.1, FDR = 2.2e-6) was *gck*: this finding is consistent with other reports that describe substantial down-regulation of *gck* expression in models of mitochondrial dysfunction [44, 45].

The remaining 115 DEGs in the continuous category belonged to the down-regulated subcategory. Here, fewer GO categories were found to contain significant enrichment; however, one such category contained phospholipase A2 inhibitors (*anxa1* and *anxa2*). A reduction in PLA2 inhibition would be consistent with the hypothesis that MET-induced cell death results in an inflammatory state. Another set of categories was involved in DNA replication/mitosis and chromatin (*irf1*, *pogz*, *med1*, *esco2*, *ppp1r10*, *hist1h1d*, *hist1h1a*, *dnmt3b*, *hellse2f2*, *mcm10*, *skp2*, *dhfr*, *cdc6 and cdk2*) (Fig 4 and S6 Table). These results were somewhat surprising given that the ablated sensory neurons are post-mitotic. There are two possibilities that could contribute to such findings- the first is that these DEG reflect changes occurring in the target cells (e.g., taste buds, skin, chemosensory cells, etc. [46–50]) of the affected neurons due to contact with degenerating axons, while a second is that mitotically active neuronal progenitors expressing nsfB are being ablated. As previous work from this lab suggested that the latter might be occurring [27, 51], additional experiments testing this possibility were performed and are discussed in the following section.

**Fig 5:**
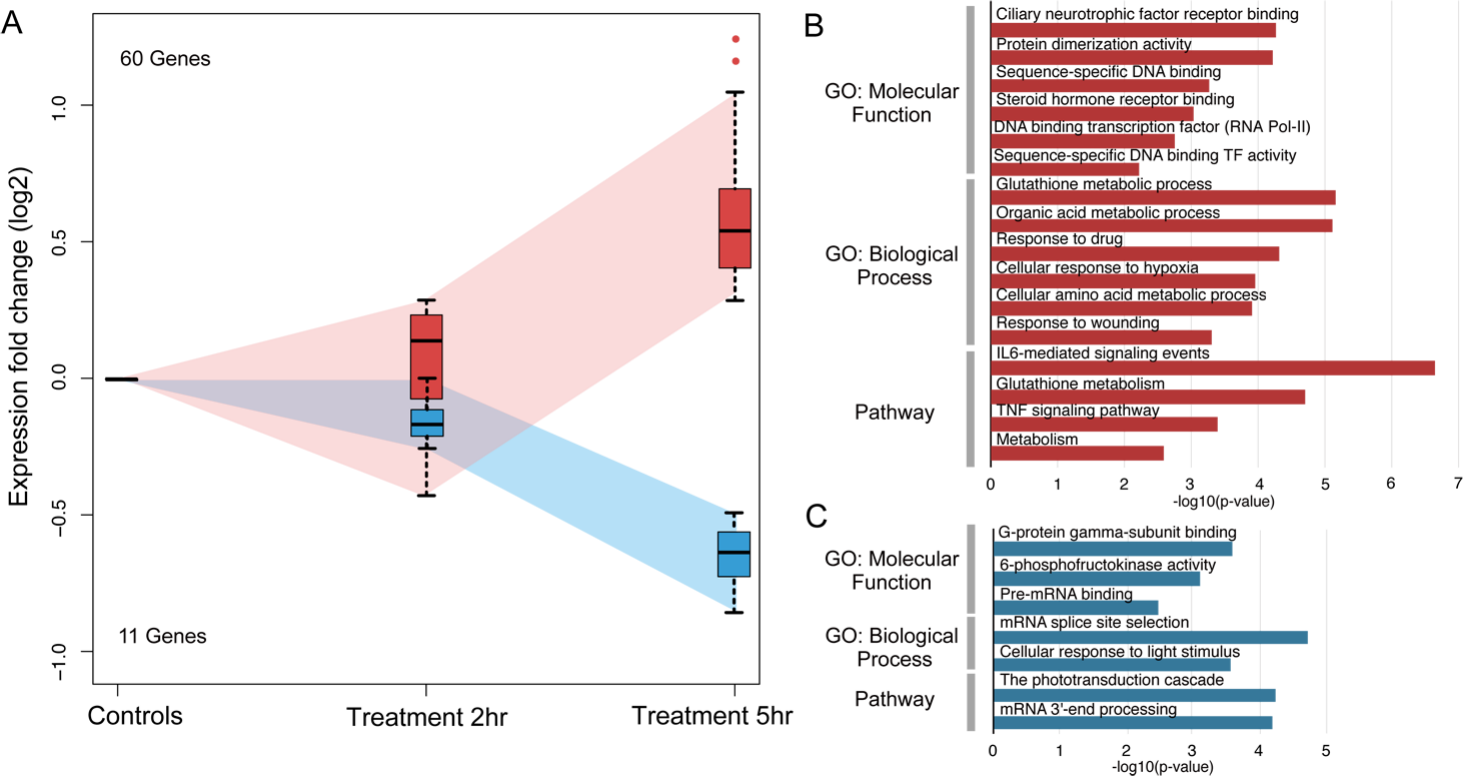
Analysis of genes found up- or down-regulated only at 5 hrs of MET-treatment. Panel A: Plot of gene expression averaged from 3 biological replicates for those DEGs found to only be differentially regulated at 5 hrs of treatment. Panel B: Representative GO categories enriched for the up-regulated genes are shown. Panel C: Representative GO categories showing enrichment for the down-regulated genes are shown. For both Panels B and C, length of bar corresponds to degree of significance (longer bars = greater significance), *p*-values determined by Benjamini-Hochberg procedure.

Finally, GO analysis of the late response DEGs (a total of 71 genes) revealed that as the larvae were exposed to MET for longer times (5 hrs), new sets of DEGs were identified that revealed enrichment in new categories such as those involved in metabolism (*cers2*, *mgst1*, *gclm*, *sgpl1*, *gls*, *papss2*, *sqrdl*, *cyp46a1*, *g6pd*, *ptgs2*, *ggt1* and *slc5a1*), transcriptional regulation (*cebpb*, *cebpd*, *bach1*, *epas1*, *cers2*, *junb*, *maff* and *stat3*), glutathione metabolism (*mgst1*, *gclm*, *g6pd* and *ggt1*) and IL-6/TNF signaling pathways (*sele*, *cebpb*, *cebpd*, *stat3*, *junb*, *creb3l3*, *tnfrsf1a* and *il6st*) (Fig 5 and S7 Table). All of these new categories are consistent with the hypothesis that as neuronal death and axon degeneration increases with time in MET, gene networks involved in inflammatory processes such as glutathione metabolism and cytokine signaling become activated. The few categories found for the down-regulated DEGs (Fig 5 and S8 Table) were primarily concerned with mRNA processing (*srsf6* and *srsf1*) and phototransduction (*sag* and *gnb5*); this latter category represents the fact that the small numbers of retinal cells expressing the 3.2:NTR transgene (data not shown) are ablated after prolonged exposure to MET.

### Subcategory of DEGs reflect neurogenesis

**Fig 6:**
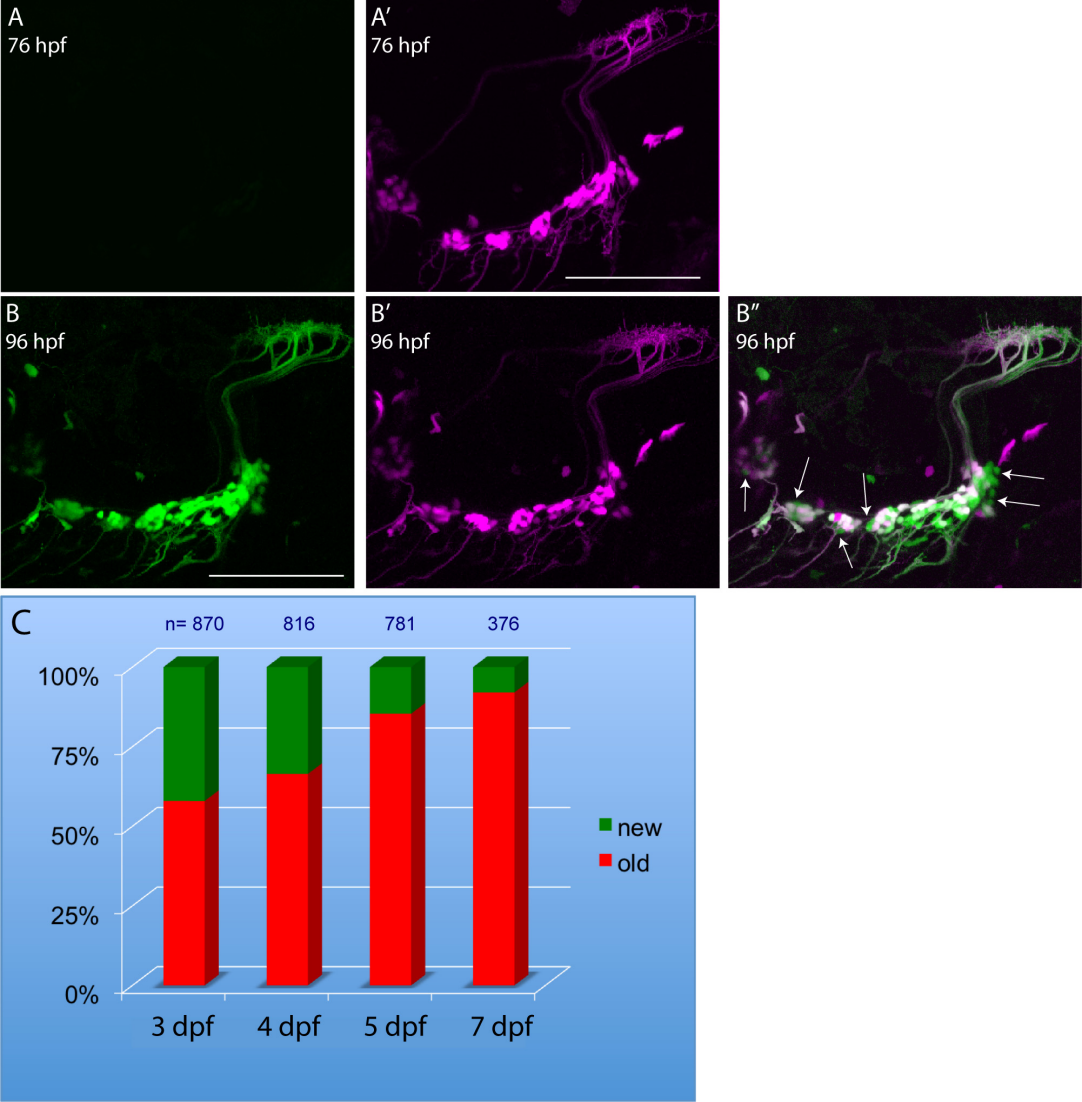
Epibranchial neurogenesis is occurring during timeframe of MET treatment. Panels A and A’ respectively, show the green and red (magenta) images of epibranchial neurons in a 76 hpf *p2rx3*.*2*^*−4*.*0*^:*gal4VP16;UAS*:*kaede* larva just after exposure to a 405nm laser: note that all kaede has been converted to red fluorescence. Panels B, B’ and B” show the same embryo 20 hours later in the green, magenta and combined channels, respectively. Only newly born neurons show green fluorescence alone (some of these neurons are indicated by the arrows in B”), whereas previously existing neurons contain both photoconverted (magenta) and newly-synthesized (green) kaede. Scale bars in A’,B = 100 μm. Panel C represents the percentage of labeled neurons (total numbers counted are shown at the tops of the bars), 24 hours after photoconversion at the indicated stage, that were either green^+^/red^-^ (newly born) or green^+^/red^+^ (pre-existing).

As previously described, there were a number of transcripts encoding proteins involved in cell cycle (*cdk2*, *dtl*, *E2F2*, *E2F3*, *skp2*, and *slbp)* and DNA replication (*cdc6*, *chtf8*, *dhfr*, *esco2*, *lig1 and mcm10)* in the continuous response category that exhibited a FC > -1.2. Earlier work from this lab demonstrated the *p2rx3*.*2* promoter fragment used also drives transgene expression in the progenitors of the epibranchial neurons [27]. Thus, the decreased numbers of transcripts for genes involved in mitosis and DNA replication might be reflective of the loss of these NTR-expressing progenitors. If this were the case, then neurogenesis of the ganglionic neurons would need to be occurring before and during the time of MET exposure. To test this possibility, we exploited the photoconvertible fluorescent reporter kaede, which has been used by others as a tool for birthdating peripheral neurons [41]. Neurogenesis was investigated in the larvae between 3–8 dpf, when the bulk of the ganglia form and complete their circuitry, and which brackets the time frame of our MET experiments [27]. Our approach allowed us to discern which neurons were present before photoconversion (contain both red and green fluorescence) versus neurons born since photoconversion (green alone). One such conversion experiment is shown in Fig 6. Fig 6A and 6A’ illustrate the ability to completely convert the green form of kaede in sensory neurons into the red form. Fig 6B–6B” show that 20 hrs later, newly biosynthesized green kaede was detectable in both old and nascent neurons in the same larva. Conversion was performed on four to eleven individual larvae for each time point, with the total number of neurons counted post-conversion ranging from 376 to 870. The results from these experiments are summarized in Fig 6C, which shows that new neurons were added to the cranial ganglia throughout the 3–6 dpf time period. The data showed that production of new sensory neurons was highest during the 3–4 dpf period, and decreased steadily thereafter, although small numbers of new neurons were still being added even at the 7–8 dpf period. Of particular relevance was that roughly 30% of existing neurons at 5 dpf were born during the time frame of the MET experiments (i.e., between 4–5 dpf). Therefore, the observed decreases in transcripts related to cell cycle and DNA replication genes could be at least partially explained by the MET-induced cell death of neuronal progenitors.

### Absence of peripheral glia alters the gene expression landscape in larvae undergoing sensory neuron degeneration

Two of the most obvious morphological changes seen in larvae after 5 hrs of MET treatment, the formation of mCherry^+^ puncta in the cranial nerves and the recruitment of macrophages to the degenerating unmyelinated sensory axons/neurons, were shown to be dependent upon the presence of peripheral glia [29]. Previous work has shown that in response to nerve injury, myelinating Schwann cells undergo a conversion from a supporting cell to a repair cell, which begins the processes of phagocytosis and inflammation [15]. This dedifferentiation/transdifferentiation is mediated by altered gene expression [14, 52]. We postulate a similar process occurs in non-myelinating glia and could account for the responses seen in our model of unmyelinated nerve injury. We propose that comparison of gene expression after 5 hrs of MET treatment in the wild-type larvae to that in treated mutant larvae would identify the gene pathways used by non-myelinating glia. To test this possibility, we carried out the next set of experiments using the *3*.*2*:*NTR;foxd3*^*zd10*^ line, in which the mutants lack peripheral glia [27, 30, 31].

cDNA libraries were prepared as previously described and subjected to Illumina sequencing to an average depth of 31 million reads for control and 35 million reads for treated replicates. The percentage of reads mapped, the number of genes identified and the percentage of reads corresponding to ribosomal RNAs were similar to those obtained with the wild-type larvae analyzed previously (S9 Table).

Bioinformatic analyses revealed a total of 529 unique DEGs in the *3*.*2*:*ntr*;*foxd3*^*-/*-^ larvae treated for 5 hrs with MET when compared to non-treated mutants (S10 Table). Of these, 292 were up-regulated and 237 were down-regulated DEG (FDR<0.05 and FC >1.2 / -1.2). The RNA biotypes were found to be primarily protein coding (503), with small numbers of other categories present: lincRNA (9), processed transcripts (8), unprocessed (3), antisense (2), miscellaneous (2), mitochondrial rRNA (1) and snoRNA (1).

**Fig 7:**
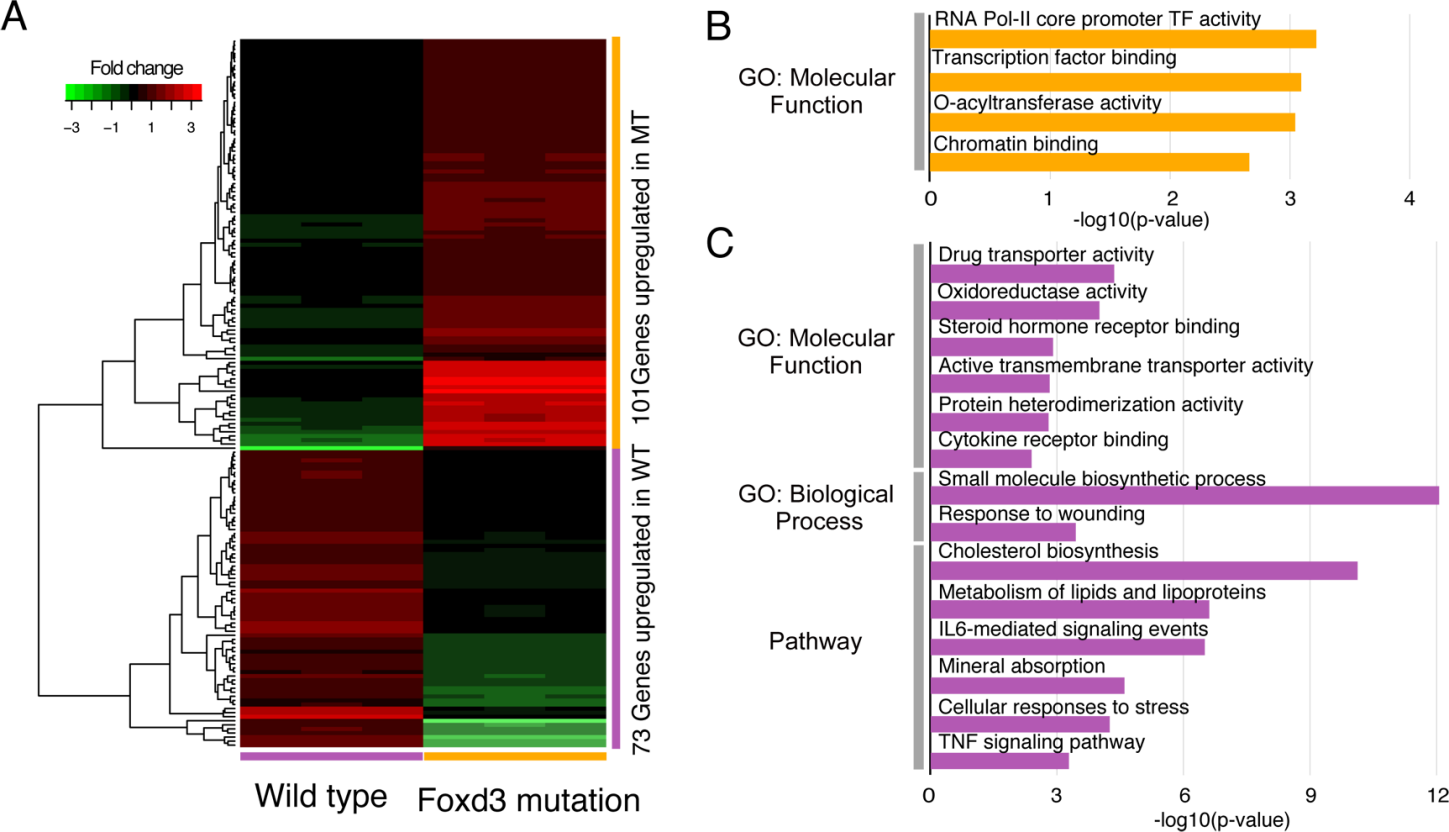
Comparison of foxd3 mutant and wild-type up-regulated DEGs found after 5 hrs of MET treatment. Panel A is a hierarchical clustering heatmap showing DEG (FDR<0.05 and FC > 1.2) found in each biological triplicate from either the wild-type or mutant transcriptomes. Gene expression fold-changes to respective controls (log_2_ ratios) are color-coded (red up-regulated and green down-regulated) Panel B: Representative GO categories enriched for the up-regulated genes found in the mutant; Panel C: representative GO categories enriched for the up-regulated genes found in the wild-type transcriptome. Length of bar corresponds to degree of significance (longer bars = greater significance), *p*-values determined by Benjamini-Hochberg procedure.

**Fig 8:**
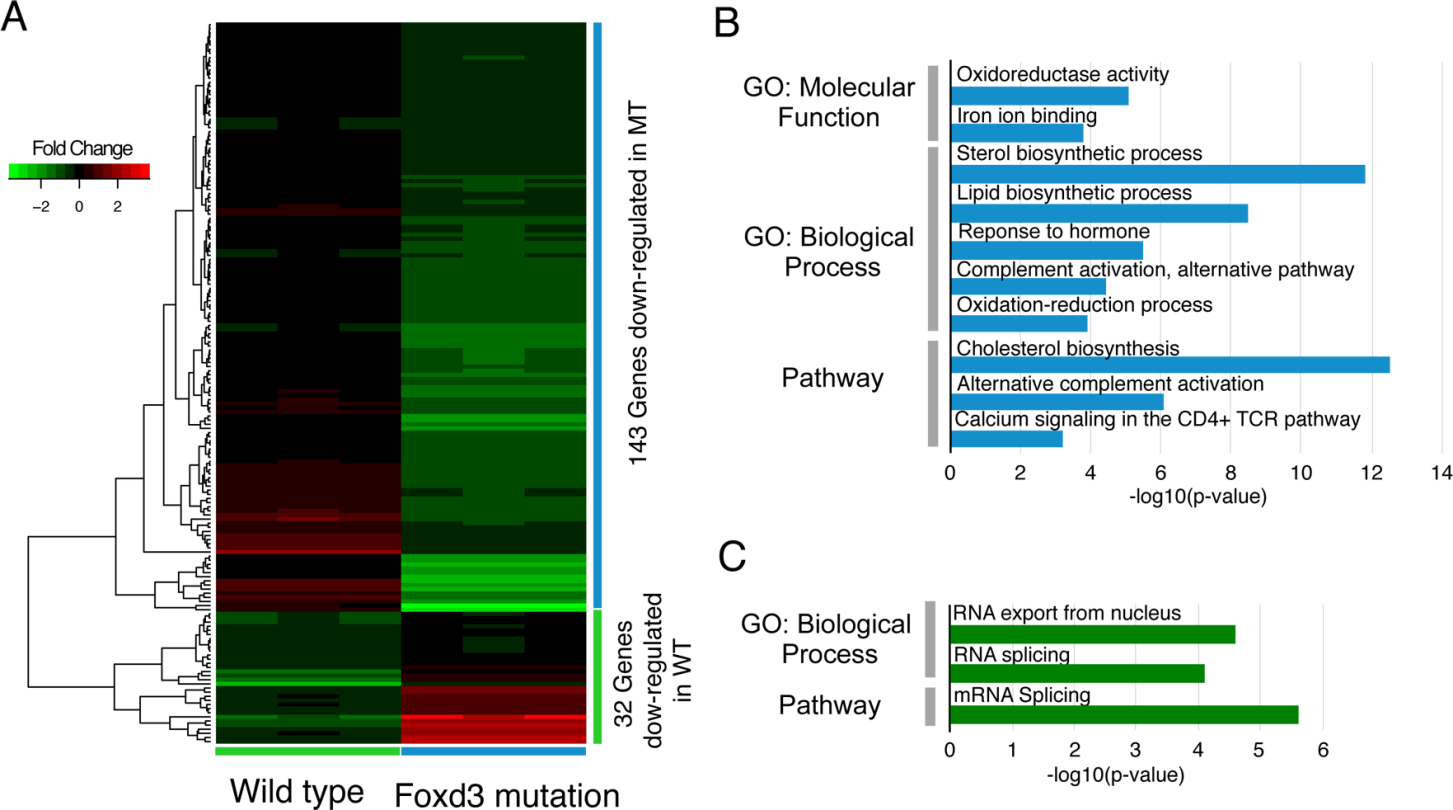
Comparison of foxd3 mutant and wild-type down-regulated DEG found after 5 hrs of MET treatment. Panel A is a hierarchical clustering heatmap showing DEG (FDR<0.05 and FC < -1.2) found in each biological triplicate from either the wild-type or mutant transcriptomes. Gene expression fold-changes to respective controls (log_2_ ratios) are color-coded (red up-regulated and green down-regulated). Panel B: Representative GO categories enriched for the down-regulated genes found in the mutant; Panel C: representative GO categories enriched for the down-regulated genes found in the wild-type transcriptome. Length of bar corresponds to degree of significance (longer bars = greater significance), *p*-values determined by Benjamini-Hochberg procedure.

Comparison of the DEGs sets from the wild-type and mutant 5 hr treatments revealed that 181 DEGs were expressed in the wild-type only and 411 DEGs in the mutant only. Given that the mutants lack peripheral glia, it is expected that the DEGs unique to the wild-type larvae contain genes involved in the glial responses to nerve damage. We used GO analysis to identify cellular processes altered in each condition in order to elucidate mechanisms that were glial dependent. A first step was to confine the pools of DEGs used for analysis to those with the least potential for false negatives (arising from biological variability across replicates that results in FDR values that fall short of the <0.05 cutoff). Therefore, DEGs chosen for analysis were limited to those that met both statistical cutoffs in one condition and did not reach the FC cutoff in the other condition: *e*.*g*., for the wild-type only up-regulated DEGs analysis, we included only those wild-type genes that met both statistical cutoffs and did not exhibit a FC >1.2 in the mutant RNA seq results. Using these criteria, 105 DEGs from the wild-type only pool (73 up-regulated and 32 down-regulated) and 244 DEGs from the mutant only pool (101 up-regulated and 143 down-regulated) were selected and used for analysis. Cluster analysis of the two DEGs pools showed that distinct and non-overlapping sets of genes were up-regulated (Fig 7A) or down-regulated (Fig 8A), indicating that the effects of MET treatment on gene expression was heavily influenced by the presence or absence of glia. When GO analysis was performed, the mutant pool showed significant enrichment in only a small number of processes for up-regulated genes, such as transcription factor and chromatin binding (*foxo1*, *hif1a*, *etv4*, *rest*, *clock*, *hif1a*, *rnf25*, *med*, *klf1*, *loxl2*, *hist1h1d* and *tp53bp2*) (Fig 7A and 7B and S12 Table), while down-regulated genes fell into categories involving oxidation-reduction (*cyp3a5*, *cox4i1*, *cyp51a1*, *tp53i3*, *hsd11b1l*, *ptgs2*, *msmo1*, *sc5d*, *dhfr*, *tm7sf2*, *dio1*, *haao*, *aldh2* and *cyb5r2*), sterol biosynthesis (*cyp51a1*, *hmgcs1*, *msmo1*, *sc5d*, *fdps*, *ebp*, *tm7sf2*, *lss* and *cyb5r2*) and alternative complement activation pathways (*cfb*, *cfd* and *c3*) (Fig 8A and 8B, and S14 Table). This was the reverse of what was seen with the wild-type pool. Here, only a small number of categories were found to be enriched in down-regulated DEGs, e.g., those involving mRNA splicing (*hnrnpa0*, *srsf1*, *srsf6 and hnrnpa1*) (Fig 8C and S13 Table). In contrast, the up-regulated DEGs showed enrichment in multiple categories (Fig 7C and S11 Table), many of which are expected to be altered in supportive cells (*i*.*e*., peripheral glia) as they respond to cytotoxic insults to the cells they assist (neurons): *e*.*g*., oxidoreductase activity (*msmo1*, *sc5d*, *cyp51a1*, *bbox1 and cyp46a1*) and metabolism/response to stress (*ipc*, *msmo1*, *sc5d*, *sgpl1*, *agt*, *ebp*, *cyp51a1*, *abcb11*, *lss*, *hmgcs1*, *cyp46a1*, *ggt1*, *cebpb*, *hist2h2be*, *hist1h4l*, *fos*, *epas1* and *stat3*). Of particular interest are the sets of genes implicated in inflammatory responses: response to wounding (*sele*, *cebpb*, *fos*, *agt*, *cd40lg*, *c3*, *atp1b1*, *f5*, *il6st*, *ggt1* and *stat3*), IL6-mediated signaling (*junb*, *cebpb*, *fos*, *il6st*, *stat3 and tnfrsf1a*) and TNF signaling (*sele*, *junb*, *cebpb*, *fos and tnfrsf1a*). The up-regulation of these genes in the wild-type only pool of DEGs is in keeping with the finding that inflammatory responses such as debris clearance and macrophage recruitment were not seen in the mutant larvae, and strongly suggest that these processes are being mediated by the peripheral glia.

**Fig 9:**
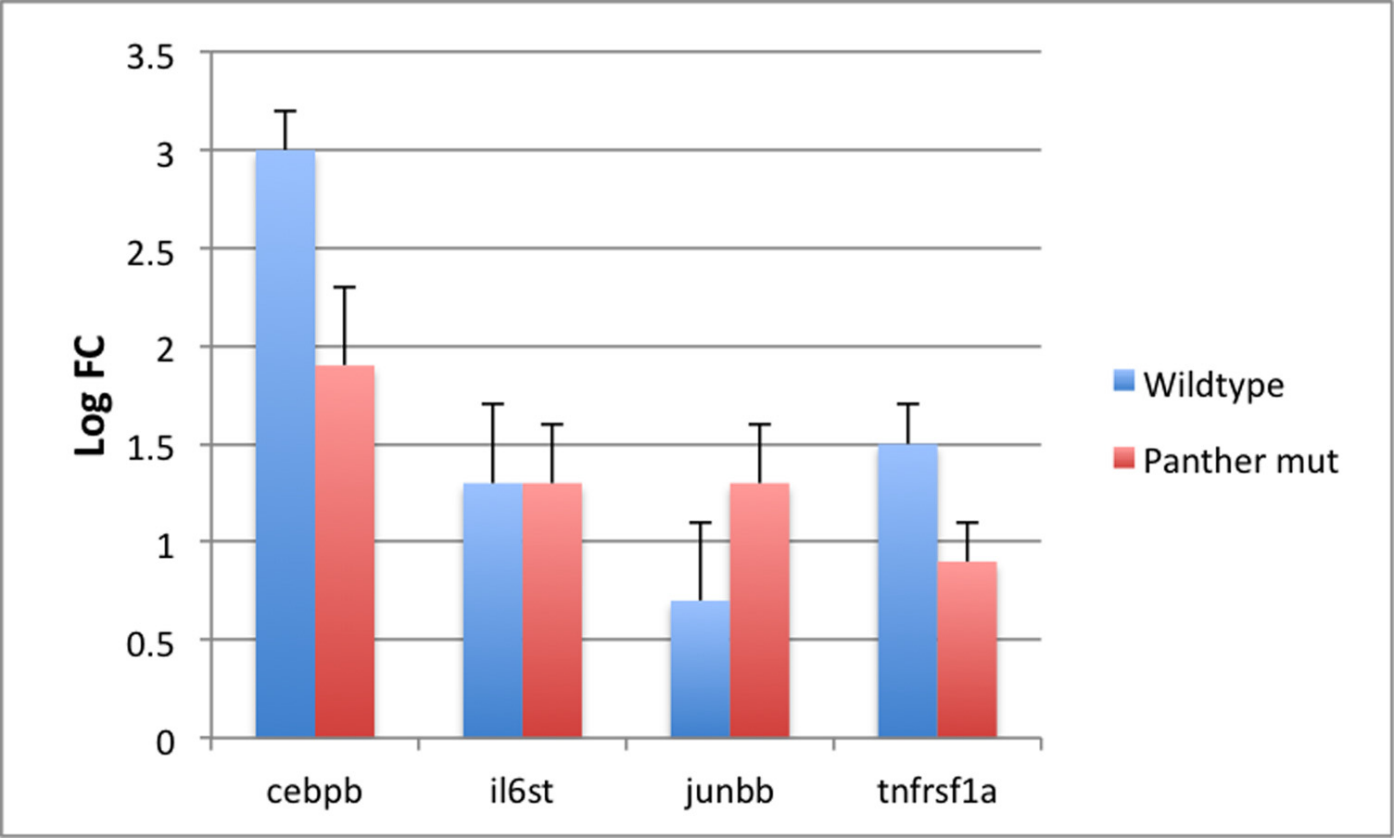
qPCR results for gene expression in wild-type and *panther* mutants. Bar graph showing qPCR determined log fold change (log FC) in expression of *cebpb*, *il6st*, *junbb*, *and tnfrsf1a* in wild type larvae (blue) and *panther (csfr1a*^*j4e1*^) mutant larvae (red). Data represent means ± S.E.M. of at least 3 experiments. Differences in mean values between conditions for each gene were not statistically significant.

It is possible that the changes in inflammatory gene expression could be accounted for by the peripheral glia or by the immune cells recruited to the sites of nerve injury. To determine if macrophages and/or microglia are contributing to the observed up-regulated inflammatory gene expression, we utilized the *panther*^*j4e*^ (*csfr1a*^*-/*-^*)* mutant line. In this line, colonization of the head by macrophages and microglia is delayed until after 4 dpf [53]. Previously, we showed that in these mutants macrophages were not recruited to the areas of MET-induced neuronal damage [29]. When transcription levels for a number of the inflammation-related DEGs were assayed by qPCR using cDNA from 5 hr MET-treated *3*.*2*:*NTR;panther*^*j4e*^ larvae versus untreated larvae, elevated expression levels similar to those seen for larvae in a wild-type background were found (Fig 9). This finding, together with the results from the foxd3 mutant work, strongly suggests that it is the peripheral glia that are the source of the increased transcription seen for these genes after 5 hrs of MET-induced neuronal damage.

These findings, together with those obtained using in vivo imaging [29], provide support for the proposition that non-myelinating glia are undergoing a conversion from a supporting, ensheathing cell phenotype to one involving active phagocytosis and recruitment of macrophages. A similar process has been more fully investigated in myelinated nerve injury. Upon nerve injury, myelinating Schwann cells stop producing myelin [54] and begin phagocytosing debris via a novel form of autophagy [16]. Schwann cells then exhibit increased expression of markers for immature Schwann cells [52] in a process that has been termed both dedifferentiation and activation, resulting in a ‘repair’ Schwann cell that is critical for axonal regeneration [14]. This phenotypic plasticity is driven by activation of signaling pathways involving p38/MAPK [55, 56] and ERK [57, 58]. Activation of those pathways leads to a rapid increase in the levels of the transcription factor *c-jun*, a known negative modulator of myelination [59]. In a subsequent study using *c-jun* mutant mice, a large number of genes normally up-regulated in injured nerves (including several involved in neuronal survival and regeneration) were not activated, there was increased death of sensory neurons in response to nerve crush, myelin clearance was delayed, axon regeneration was impaired and functional nerve recovery failed [15]. These findings led the authors to propose that *c-jun* acts as a ‘master regulator’ of Schwann cell reprogramming in response to axonal injury. We did not observe any changes in *c-jun* transcription in our study, which suggests the possibility that myelinating and non-myelinating glia might respond to axonal damage differently.

This idea is supported by several studies investigating the molecular responses of non-myelinating glial to nerve injury. Cheepudomwit et al. [60] reported that levels of RNA encoding the cytokines MCP, IL-2, IL-6 and IL-10, IL-1ß and LIF RNA were increased in the sciatic nerve when it was transected (affecting both unmyelinated and myelinated nerves axons), but that the increases in sciatic IL-1ß and LIF RNAs were not seen when unmyelinated axons were selectively damaged using capsaicin injections. Additional indirect support comes from studies demonstrating differences in proliferative responses of non-myelinating versus myelinating glia in motor nerve injury and in growth factor expression after nerve root denervation/grafting [61–63].

While c-jun expression was unaltered in our model, a number of other transcription factors (TF) did show changes in the wild-type at both 2 hrs (*cbx7a*, *ddx26b*, *dedd1*, *dtl*, *e2f2*, *e2f3*, *egln3*, *egr4*, *fboxo15*, *fosl1a*, *hbp1*, *hif1al*, *ier2*, *klf9*, *klf13*, *klf16*, *klf17*, *nr0b2a*, *nr0b2b*, *nr1i2* and *rxrab*) and at 5 hrs (*agf2*, *bzw1b*, *cbx7a*, *cebpb*, *cebpd*, *ddx26b*, *egln3*, *fosl1a*, *hif1al*, *hig1*, *junbb*, *klf9*, *klf13*, *nr0b2b* and *nr1i2*) of MET-treatment. These most likely underrepresent the total number of TF that are affected, as it is highly likely that additional TF are present among the numerous uncharacterized genes present in each of the DEGs pools. The incomplete overlap between these two groups of TF most likely explains the differences in the panels of DEG seen at the two timepoints.

Involvement of IL6 and TNF signaling pathways in responses to axonal injury/degeneration is not unexpected, as previous studies have implicated these pathways as being activated in various models of peripheral nerve injury [60, 64–66]. However, as both sensory neurons and peripheral glia express the genes for multiple cytokines, chemokines and their receptors, including IL-6 and its co-receptor proteins il6st (gp130) and il6r [64, 67] it remains to be determined if the signaling is from neuron to glia, glia to glia or glia to neuron, or a combination thereof.

This is the first transcriptome study of metronidazole-induced neuronal death in zebrafish and the response of non-myelinating glia to sensory neuron degeneration. As the appropriate tools become available, future investigation into specific genes and cellular processes in selected subpopulations of cranial cells will provide a deeper understanding of how the non-myelinating glial response to axonal and soma degeneration leads to activation of the pro-inflammatory pathways underlying responses to nerve injury.

## Supporting Information

S1 Fig Spearman Correlation Matrix. (TIFF)

S1 Table Sequencing summary for wild-type larvae. (XLSX)

S2 Table DEG in wild-type at 2 hrs treatment. (XLSX)

S3 Table DEG in wild-type at 5 hrs treatment. (XLSX)

S4 Table GO analysis of early response up-regulated DEG. (XLSX)

S5 Table GO analysis of continuous response up-regulated DEG. (XLSX)

S6 Table GO analysis of continuous response down-regulated DEG. (XLSX)

S7 Table GO analysis of late response up-regulated DEG. (XLSX)

S8 Table GO analysis of late response down-regulated DEG. (XLSX)

S9 Table Sequencing summary for mutant larvae. (XLSX)

S10 Table DEG in mutant at 5 hrs treatment. (XLSX)

S11 Table GO analysis of wild-type only vs. mutant up-regulated DEG. (XLSX)

S12 Table GO analysis of mutant only vs. wild-type up-regulated DEG. (XLSX)

S13 Table GO analysis of wild-type only vs. mutant down-regulated DEG. (XLSX)

S14 Table GO analysis of mutant only vs. wild-type down-regulated DEG. (XLSX)
