# Supplementary figures and images for: Transcriptome Analysis of Chemically-Induced Sensory Neuron Ablation in Zebrafish
Source: PLoS One. 2016 Feb 10;11(2):e0148726. doi: 10.1371/journal.pone.0148726 (PMC4749159; doi:10.1371/journal.pone.0148726)

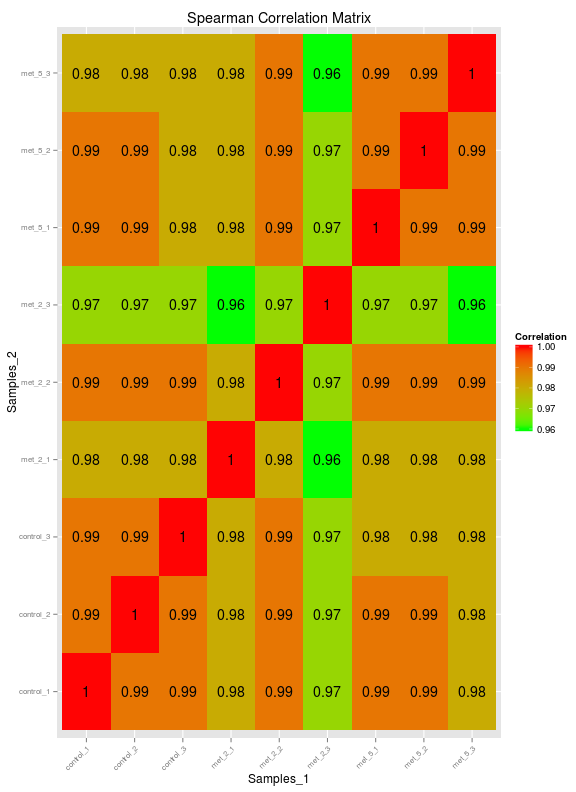

Supplement: S1 Fig — (TIFF) [file pone.0148726.s001.tiff]
